# 

**Published:** 1895-09-21

**Authors:** 


### CONTENTS.

Nurses, their Uniform and the Public - - - - 423

The Memorial to Sir Andrew Clark, Bart. - - - 424

The Symptoms of Aneurism. By A. PEARCE GOULD, F.R.C.S. 425

Social Problems.—Child Life in London - - - - - 426

Annotations.—Discredited Hospitals—Polygamy and Progress—Buried Alive - - - - - - - - - - - 427

Medical Progress and Hospital Clinics—

PERITYPHLITIS. - - - - - - - - - - - 429

PROGRESS IN SURGERY.—Brain Surgery—Surgery of the Intestines 431

NEW APPLIANCES AND THINGS MEDICAL - - - - - 434

Notes and News - - - - - - - - - - - 428

The Institutional Workshop—

WITHIN THE HOSPITALS.—Boscombe Hospital and Provident Dispensary - - - - - - - - - - - - 435

HOSPITAL CONSTRUCTION.—Progress in German Hospitals - - 4‧5

PRACTICAL DEPARTMENTS.—The "Sister Louise" Ice Cup - - 436

EXHIBITION BY THE DRUG, CHEMICAL, AND ALLIED TRADES - 436

Editor's Letter-box - - - - - - - - - - 437

Practical Points - - - - - - - - - - 437

Hospital Saturday in London—Scraps and Gleanings 437

EXTRA SUPPLEMENT.—THE NURSING MIRROR.

News from the Nursing World.—Taking Temperatures—Volunteered Approbation—Grateful Patients—Kind and Considerate—An Exhibition of Nursing Appliances—Registration in Sydney — An Asylum Adventure — Prize-giving at the London Hospital—A Tribute to Sister Augustine—Helping the Helpless—Superstitious—Incurable, but not Inconsolable—A Hospital for Infantile Diarrhœa—Bridgwater District Nurse Association - - - - - - - - - - clxv

New York City Training School for Nurses - - clxviii

Appointments - - - - - - - - - - - clxviii

Elementary Physiology for Nurses. By C. F. MARSHALL, M.D., B.Sc., F.R.C.S. VII.—The Respiratory and Excretory System - - - - - - - - - - clxvii

Minor Appointments - - - - - - - - - clviii

Everybody's Opinion - - - - - - - - - clxix

Wants and Workers - - - - - - - - - clxix

Nuns or Nurses? - - - - - - - - - clxix

Novelties for Nurses - - - - - - - - clxix

Notes and Queries - - - - - - - - - clxx

For Reading to the Sick - - - - - - - - clxx

